# Knowledge of stroke risk factors among primary care patients with previous stroke or TIA: a questionnaire study

**DOI:** 10.1186/1471-2296-11-47

**Published:** 2010-06-15

**Authors:** Andrzej Sloma, Lars G Backlund, Lars-Erik Strender, Ylva Skånér

**Affiliations:** 1 Centre for Family and Community Medicine (CeFAM), Karolinska Institutet, Alfred Nobels allé 12, SE-141 83 Huddinge, Sweden

## Abstract

**Background:**

Survivers of stroke or transient ischaemic attacks (TIA) are at risk of new vascular events. Our objective was to study primary health care patients with stroke/TIA regarding their knowledge about risk factors for having a new event of stroke/TIA, possible associations between patient characteristics and patients' knowledge about risk factors, and patients' knowledge about their preventive treatment for stroke/TIA.

**Methods:**

A questionnaire was distributed to 240 patients with stroke/TIA diagnoses, and 182 patients (76%) responded. We asked 13 questions about diseases/conditions and lifestyle factors known to be risk factors and four questions regarding other diseases/conditions ("distractors"). The patients were also asked whether they considered each disease/condition to be one of their own. Additional questions concerned the patients' social and functional status and their drug use. The t-test was used for continuous variables, chi-square test for categorical variables, and a regression model with variables influencing patient knowledge was created.

**Results:**

Hypertension, hyperlipidemia and smoking were identified as risk factors by nearly 90% of patients, and atrial fibrillation and diabetes by less than 50%. Few patients considered the distractors as stroke/TIA risk factors (3-6%). Patients with a family history of cardiovascular disease, and patients diagnosed with carotid stenosis, atrial fibrillation or diabetes, knew these were stroke/TIA risk factors to a greater extent than patients without these conditions. Atrial fibrillation or a family history of cardiovascular disease was associated with better knowledge about risk factors, and higher age, cerebral haemorrhage and living alone with poorer knowledge. Only 56% of those taking anticoagulant drugs considered this as intended for prevention, while 48% of those taking platelet aggregation inhibitors thought this was for prevention.

**Conclusions:**

Knowledge about hypertension, hyperlipidemia and smoking as risk factors was good, and patients who suffered from atrial fibrillation or carotid stenosis seemed to be well informed about these conditions as risk factors. However, the knowledge level was low regarding diabetes as a risk factor and regarding the use of anticoagulants and platelet aggregation inhibitors for stroke/TIA prevention. Better teaching strategies for stroke/TIA patients should be developed, with special attention focused on diabetic patients.

## Background

More than 30 000 patients suffer from stroke and 8 000 suffer from transient ischaemic attacks (TIA) in Sweden annually [1]. Survivors of stroke or TIA remain at high risk of new vascular events [2-4]. Regardless of this fact, many reports have shown that secondary prevention after stroke or TIA is not satisfactory [5-7]. One of the reasons for unsatisfactory secondary prevention could be patients' lack of knowledge about risk factors for suffering from new events of stroke, which was suggested as a contributing factor to the lack of compliance with medical advice and treatment [8]. A number of previous studies have assessed knowledge in the general population concerning stroke, its symptoms and risk factors. Most of those studies have demonstrated poor understanding of stroke risks and symptoms among people in general [9-12]. Other studies have shown that knowledge about stroke and stroke risk factors was poorest among groups at highest risk of suffering from stroke [13,14]. Further, a few previous studies assessing stroke or TIA patients' knowledge about stroke risk factors have indicated poor knowledge about stroke, including knowledge about risk factors some months after stroke [7,15], in rehabilitation patients [16,17] or in an Indian context [18]. However, the extent to which increased knowledge about stroke can be translated into improved patient recovery and adjustment remains unclear [19].

Our objective was to study primary health care patients who have already suffered from stroke or TIA (referred to in the following as stroke/TIA) regarding their knowledge about risk factors for having a new event of stroke/TIA, possible associations between patient characteristics and patients' knowledge about risk factors, and patients' knowledge about their own treatment for stroke/TIA prevention.

## Methods

### Study design

A cross-sectional postal questionnaire study.

### Setting

Gustavsberg Primary Health Care Centre (GPHCC) is a large primary health care unit serving the majority of the population of the municipality of Värmdö, Sweden, with approximately 35 000 inhabitants within the catchment area. The population is growing and during the last ten years it has increased with 40 percent, which is the highest increase rate in Sweden during this period. Värmdö is situated in the Stockholm archipelago and the population is somewhat younger than the average Swedish population; only 10% of the inhabitants are 65 years or older (17% in Sweden). One third of the population has education from above the upper secondary school (34% in Sweden).

### Study population

The study population consisted of patients who had the diagnosis of stroke or TIA registered in the medical records at GPHCC (ProfDoc™) by May 1, 2005. We used two different softwares (Xtractor™ and Rave™) to search for stroke and TIA diagnosis codes according to the Swedish primary health care version of ICD-10 [20]: I61 (intracerebral haemorrhage), I63 (cerebral infarction), I64 (stroke, not specified as haemorrhage or infarction), I67 (other cerebrovascular diseases), I69 (sequelae of cerebrovascular disease), and G45 (transient cerebral ischaemic attacks). The diagnosis of subarachnoid haemorrhage (I60), which could be considered as a stroke subtype, was excluded due to different aetiology and risk factors [1,21]. Another source of patient identification was a separate stroke register created at GPHCC through previous searches in the medical records and through collaboration between the medical staff at GPHCC and Stockholm Söder Hospital's Stroke Care Unit. The hospital reported all patients with stroke/TIA living in Värmdö (based on postcode numbers) to GPHCC. The majority of patients fulfilling the inclusion criteria were found by using both the medical records and the separate stroke register. In total, 383 patients were identified.

**Figure 1:**
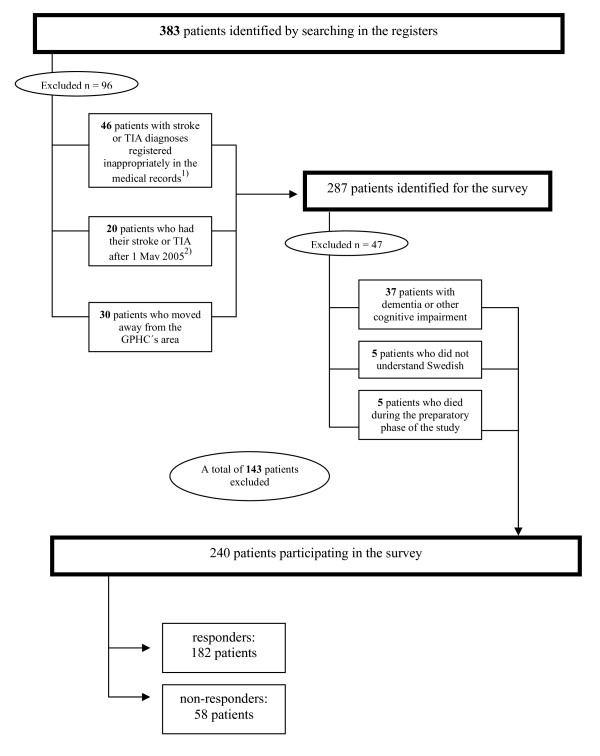
**Patients' inclusion in the study and the response rate**. Patients' inclusion in the study and the response rate. Inappropriately diagnosed patients: ^1) ^30 patients suffered from a condition other than stroke/TIA, although they initially had got this diagnosis registered; six patients suffered from traumatic brain haemorrhage, eight patients from subarachnoid haemorrhage (and were registered as I64, stroke not specified), and two patients from transient global amnesia (which is not a subtype of stroke/TIA). ^2) ^Excluded in order to make it possible to collect data from the records during a longer period and also to exclude patients still in a rehabilitation phase.

We excluded 143 patients from further investigation due to incorrectly registered diagnoses, cognitive impairment, etc. (see Figure 1). Consequently, the study population consisted of 240 patients.

### Questionnaire

A questionnaire was used to assess the patients' knowledge about diseases and conditions established as important factors increasing the risk of having a new stroke/TIA. The questionnaire was designed especially for the purpose of this study. It was based on a literature review of previous studies concerning patients' knowledge about stroke/TIA and stroke/TIA risk factors [11,12,22,23].

We tested the questionnaire in a pilot study with a sample of five persons who were not a part of the study population (staff members at GPHCC). A few changes in the wording of questions were made as a result of the pilot study.

Patients were asked to evaluate how 13 diseases/conditions, established as stroke/TIA risk factors, influenced the risk of having a new stroke/TIA [23,24]. The risk factors were presented in the questionnaire in the following order: higher age, hyperlipidemia, diabetes, a family history of cardiovascular disease, atrial fibrillation, hypertension, overweight, regular physical exercise, excessive alcohol consumption, previous stroke/TIA, carotid stenosis, smoking and ischaemic heart disease. The questionnaire was designed as a series of questions about stroke/TIA risk factors with the same response alternatives. A common problem with this kind of questionnaire is that after being asked a series of similar questions, some people may give the same answer to each question without really considering it. To reduce this risk, four questions regarding medical diagnoses/conditions which are known not to be stroke/TIA risk factors ("distractors") were added: rheumatoid arthritis, osteoporosis, thyroid disease and allergy. The distractors were placed at random in the questionnaire.

An example of a question was: "How do you think that diabetes influences the risk of having a new stroke/TIA?" Possible answers were: "it increases the risk", "it reduces the risk", "it does not influence the risk", "do not know". We considered "increases the risk" to be the correct answer for questions about stroke/TIA risk factors, except for the question about regular exercise habits, where "reduces the risk" was considered to be correct. The correct answer for the distractors was "does not influence the risk".

For all items except higher age and previous stroke/TIA, the patients were also asked if they regarded the disease/condition in the questionnaire as their own; for example, "Do you have diabetes?" with the following response alternatives: "yes", "no", "do not know". For questions concerning lifestyle factors (overweight, level of physical activity, alcohol consumption, smoking) we asked the patients to add self-reported information to their answers about their weight and height (from which Body Mass Index, BMI was calculated), exercise habits, alcohol and tobacco use. Seven questions about the patients' social and functional status were also added. Finally the patients were asked to list all the drugs they were taking and to mark those which they considered to be prescribed for preventing new events of stroke/TIA.

The questionnaire also included an open-ended question, where the patients were asked to give some examples of other conditions or diseases that they thought could influence the risk of having a new stroke/TIA.

### Background information about the patients

Data concerning age, sex, stroke/TIA diagnoses, and number of years since the first ever stroke/TIA event were available for all patients in the study population who returned the questionnaire and these were collected from the medical records during the preparatory phase of our study. All stroke/TIA patients living in GPHCC's catchment area had been invited annually to group meetings where patients and their relatives had an opportunity to get information about stroke/TIA risk factors and their treatment. The patients own risk factors were always discussed according to a checklist, which included blood pressure, blood lipids, blood glucose and lifestyle factors. Data about participation in these group meetings were also included as background data for patients in the study population.

### Data analyses

The main outcome measure of the study was the extent to which patients could correctly identify the different stroke/TIA risk factors in the questionnaire. Only the answer "it increases the risk" was considered to be correct (except for the question about regular exercise habits, which was constructed in the opposite way: "How do you think that regular exercise influences the risk of having a new stroke or TIA?" and where the correct answer was "it reduces the risk").

We also calculated each patient's knowledge about stroke/TIA risk factors, defined as the number of correctly identified stroke/TIA risk factors in the questionnaire (range 0-13).

The patients' knowledge about their own treatment for stroke/TIA prevention was assessed by reviewing the patients' ability to mark, in their self-reported lists, the drugs which they thought were prescribed to prevent new events of stroke or TIA.

### Statistics

In the analysis of differences between patient groups (for example, between responders and non responders, between genders, etc.), we used the t-test for continuous variables and the chi-square test for categorical variables. For continuous variables, means ± standard deviation (SD) are presented.

For analysis of the relation between patients' knowledge about stroke/TIA risk factors and other variables, an ordered logistic regression analysis was performed. Spearman's rank correlation coefficients were first calculated between the outcome variable, described as the number of correctly identified stroke/TIA risk factors in the questionnaire for each patient, and each one of the other variables (age, sex, stroke/TIA related diagnoses, lifestyle factors, participating in stroke group activities, educational level and information about social and functional status). Four variables were selected (age, heredity for stroke/TIA, occurrence of atrial fibrillation and diagnosis of cerebral haemorrhage) for which p-values for the Spearman rank correlation were significant (p < 0.05).

The dependent variable (the number of correctly identified risk factors) was then categorized for the purpose of statistical analysis. We defined "good" and "poor" knowledge of stroke/TIA risk factors according to the distribution of percentiles of correct answers. This resulted in cut-off points of less than 8 correct answers (below the 25% percentile), which was categorized as poor knowledge (45 patients); and more than 11 correct answers (above the 75% percentile), which was categorized as good knowledge (41 patients). The group with an intermediate number of correct answers (8-11) was categorized as having "moderate" knowledge (96 patients).

The model was adjusted by stepwise inclusion of the remaining independent variables into the model and evaluating how this influenced the variables within the model. Finally, a regression model with five variables was created (age, heredity for stroke/TIA, occurrence of atrial fibrillation, diagnosis of cerebral haemorrhage and living alone; the model is presented as adjusted for age). Statistical analyses were performed using STATA, version 9.2.

### Ethics

The study was approved by the Regional Ethics Committee in Stockholm, (file 2005/1445-31/4).

## Results

### Response rate

After one written reminder, 182 questionnaires (75.8% of a total 240) were returned.

### Non responders

For the patients who did not return the questionnaires (n = 58), we analysed available data about age, sex, time since the first stroke/TIA event, and participation in group meetings for stroke/TIA patients at GPHCC. The response rate was higher among women (83.7%) than among men (70.4%), p = 0.018. Responding women were younger (mean age 71.5 years) than non responding women (78.3 years), p = 0.021. Among responders, 50.5% had participated in group meetings for stroke/TIA patients at GPHCC compared with 20.7% of non responders (p < 0.001). Other observed differences between responders and non responders were not statistically significant.

### Study population

Mean age for all patients in the study population was 71.3 (± 9.8) years, and 82 patients (45.1%) were women. Mean time from the first ever stroke/TIA event was 6.7 (± 4.3) years. A stroke/TIA diagnosis was registered in the medical records as haemorrhagic or ischaemic stroke for 72 patients (39.6%; haemorrhagic 8.8% and ischemic 30.8%). TIA as the only diagnosis was registered for 39 patients (21.4%). For the remaining 71 patients (39%) we could not determine the stroke type by searching only for diagnostic codes because diagnoses were registered as I64, I67 or I69 (i.e. stroke not specified as haemorrhage or ischaemic, other cerebrovascular diseases, or sequelae of cerebrovascular disease). However, for most of the patients the diagnosis could be determined by reviewing the medical records for further information. In Table 1, the stroke related diagnoses based on both ICD-10 codes and other information from the medical records are shown.

**Table 1:** Stroke/TIA diagnoses according to medical records and lifestyle and social factors according to self-reported data.

	Number of patients (%) n = 182
**Stroke related diagnoses (ICD-10 codes)**	
Intracerebral haemorrhage (I61)	25 (13.7)
Cerebral infarction (I63)	103 (56.6)
Stroke, not specified as haemorrhage or infarction (I64)	6 (3.3)
Transient ischaemic attacs, TIA (G45)	48 (26.4)
**Lifestyle factors**	
Overweight (Body Mass Index, BMI) ≥ 25 kg/m^2^	59 (32.4)
Excessive alcohol consumption ^1)^	15 (8.2)
Smokers	23 (12.6)
Former smokers	78 (42.9)
Regular physical activity ^2)^	135 (74.2)
**Social factors**	
Born in Sweden	144 (79.1)
Still working	15 (8.2)
Living alone (12 men, 26 women; p = 0.009)**	38 (20.9)
Completed upper secondary education or a higher educational level (37 men, 15 women; p = 0.036)*	51 (28.0)
Needing help with personal hygiene (such as going to the toilet, getting dressed and undressed)	9 (4.9)
Needing at least one technical aid for walking/transportation: cane (27 (14.8); walker 22 (12.1); crutch 17 (9.3); wheelchair 11 (6.0)	55 (30.2)

Stroke/TIA diagnoses according to ICD-10 codes and other information from the medical records; lifestyle factors and social factors according to self-reported data.

Statistical significance at level: *) p < 0.05; **) p < 0.01; ***) p < 0.001.

^1 ^Defined in the questionnaire as drinking more than two glasses of alcohol daily (one glass equivalent to 33 cl strong beer, one glass of wine or 4 cl of spirits).

^2 ^Defined as walking or physical exercise, for example, at least a few times a week.

Lifestyle factors are presented in Table 1. Statistically significant differences between sexes were observed for BMI (women 24.7 ± 4.4 kg/m^2^, men 27.0 ± 4.4 kg/m^2^, p < 0.001) and alcohol consumption (12 men, 12.0%, and 3 women, 3.7%, reported drinking more than two standard drinks daily, p = 0.036). Social factors are also listed in Table 1. A higher proportion of the women were living alone and a higher proportion of men had a high educational level. The majority of the patients (n = 145, 79.7%) reported being able to fill in the questionnaire without any help from other persons. Ninety-two patients in the study population (50.5%) had participated in group meetings for stroke/TIA patients at GPHCC. More than half of those who had participated (52.2%) had done so more than once.

### Patients' identification of stroke/TIA risk factors

The risk factors that were most often identified by the patients (by close to 90%) were hypertension, hyperlipidemia and smoking (Table 2). Atrial fibrillation and diabetes were identified by less than 50% of the patients. The proportion of incorrect answers, i.e. where the patients answered that the risk factor "does not influence" or "reduces" the risk (for exercise habits "increases" instead of "reduces"), was generally low (under 10%). The rate of missing answers was low as well (Table 2).

**Table 2:** Proportion (%) of patients considering diseases/conditions to be or not to be stroke/TIA risk factors.

Condition/disease	Answers in the questionnaire n = 182				
	Increases the risk	Reduces the risk	No influence on risk	Do not know	Missing answers
**Stroke/TIA risk factors**					

Hypertension	87.4	0.5	2.2	7.1	2.7
Hyperlipidemia	87.4	0.5	4.4	7.1	0.5
Smoking	87.4	0.5	2.7	8.8	0.5
Regular exercise^1)^	0	80.8	9.9	7.1	2.2
Overweight	77.5	0	3.3	15.9	3.3
Ischaemic heart disease	70.9	0.5	1.1	25.8	1.6
Older age	69.8	0.5	9.3	19.8	0.5
Excessive alcohol consumption	69.2	2.2	4.4	22.0	2.2
Family history of cardiovascular disease	64.3	0.5	6.6	26.9	1.6
Carotid stenosis	63.2	0	2.7	33.5	0.5
Suffering previous stroke/TIA	62.1	1.6	6.5	27.5	2.2
Atrial fibrillation	49.5	0	5.5	42.9	2.2
Diabetes	41,8	0.5	7.1	49. 5	1.1

**Distractors**					

Osteoporosis	2.7	0	28.0	67.6	1.6
Allergy	3.3	0.5	21.4	73.6	1.1
Rheumatoid arthritis	4.4	0	14.3	80.8	0.5
Thyroid disease	6.0	0	12.6	79.7	1.6

Proportion (%) of patients considering diseases/conditions to be or not to be stroke/TIA risk factors. The conditions/diseases are presented according to the proportion of correct answers.

1) This question was constructed in an opposite way compared to the other questions: "How do you think that regular exercise influences the risk of having a new stroke/TIA?" where the correct answer was "It reduces the risk".

Only a few patients considered medical conditions that were not stroke/TIA risk factors (distractors) as stroke/TIA risk factors (Table 2). The most common answer was "do not know", although there was a group of patients who knew that these conditions did not affect the risk of having a new event of stroke/TIA (28.0% for osteoporosis, 21.4% for allergy, 14.3% for rheumatoid arthritis and 12.6% for thyroid disease). Knowledge about distractors was not correlated with knowledge about known risk factors (Sperman's rho 0.06).

**Figure 2:**
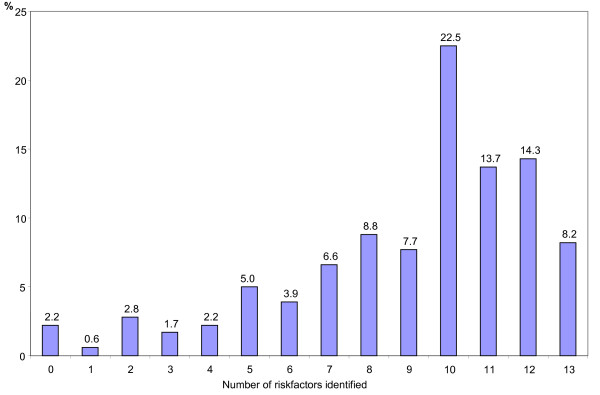
**Proportion of patients who could correctly identify different numbers of stroke/TIA risk factors**. Proportion of patients who could correctly identify different numbers of stroke/TIA risk factors.

The median number of correctly identified stroke/TIA risk factors in this group of patients was 10; 58.7% of the patients could identify 10 or more risk factors. A frequency histogram with the proportions of patients who correctly identified different numbers of stroke/TIA risk factors is shown in Figure 2.

Half of the patients in the study population had attended group meetings for stroke/TIA patients at GPHCC at least once. However, we did not find that having attended group meetings influenced patients' knowledge about stroke/TIA risk factors (data not shown).

### Associations between patient characteristics and patients' knowledge about risk factors

Over 90% of patients who had, or were treated for, hypertension, hyperlipidemia, carotid stenosis or atrial fibrillation could identify those conditions as stroke/TIA risk factors (Table 3). Patients suffering from ischaemic heart disease or diabetes identified these conditions as stroke/TIA risk factors less frequently (ischaemic heart disease 76% and diabetes 72%). Only 60% of patients who considered their alcohol consumption as high identified this as a stroke/TIA risk factor.

**Table 3:** Different risk perceptions among patients considering the disease/condition as their own.

Patients considering the disease/condition as their own/not their own			Patients who *also *identified the disease/condition as a risk factor	p-value^1)^
		n = 182 (%)	n = first column (%)	
Hypertension	Own factor	123 (67.6)	111 (90.2)	0.536
	Not own factor	53 (29.1)	46 (86.8 )	
Hyperlipidemia	Own factor	96 (57.2)	88 (91.7)	0.196
	Not own factor	65 (35.7)	59 (90.8)	
Smoking	Own factor	23 (12.6)	20 (87.0)	0.995
	Not own factor	79 (43.4)	68 (86.1)	
No regular exercise	Own factor	44 (24.2)	33 (75.0)	0.764
	Not own factor	135 (74.2)	112 (83.0)	
Overweight	Own factor	59 (32.4)	52 (88.1)	0.351
	Not own factor	114 (62.6)	87 (76.3)	
Ischaemic heart disease	Own factor	42 (23.1)	32 (76.2)	0.557
	Not own factor	130 (71.4)	91 (70.0)	
Excessive alcohol consumption	Own factor	15 (8.2)	9 (60.0)	0.738
	Not own factor	162 (89.0)	116 (71.6)	
Family history of cardiovasc. disease	Own factor	87 (47.8)	74 (85.1)	0.0006***
	Not own factor	75 (41.2)	34 (45.3)	
Carotid stenosis	Own factor	23 (12.6)	22 (95.7)	0.029*
	Not own factor	126 (69.2)	75 (59.6)	
Atrial fibrillation	Own factor	32 (17.6)	29 (90.6)	0.0005***
	Not own factor	125 (68.7)	48 (38.4)	
Diabetes	Own factor	36 (19.8)	26 (72.2)	0.013*
	Not own factor	144 (79.1)	50 (34.7)	

Different risk perceptions among patients considering the disease/condition as their own and patients not considering the disease/condition as their own. Missing answers not shown.

1) Chi square test: patients identifying the disease/condition as a riskfactor among those having it as own factor against patients identifying the disease/condition as a riskfactor among those *not *having it as own factor. Statistical significance at level: *) p < 0.05; **) p < 0.01; ***) p < 0.001.

Patients who reported that they had a family history of cardiovascular disease, carotid stenosis, atrial fibrillation or diabetes had statistically significant better knowledge about the fact that this disease/condition was a stroke/TIA risk factor compared to patients who reported that they did not suffer from these conditions (Table 3).

On the other hand, patients who reported hypertension, hyperlipidemia, smoking, lack of regular physical activity, overweight, excessive alcohol consumption or ischaemic heart disease as their own risk factors did not have statistically significant better knowledge about the fact that these diseases/conditions were stroke/TIA risk factors compared to patients who reported that they did not have these diseases/conditions. Older patients (75 years or older) did not have significantly better knowledge about age being a stroke/TIA risk factor as compared to younger patients (data not shown).

When asked to name other conditions or diseases that could increase the risk of having a new stroke/TIA, 27 patients (14.8%) mentioned stress. Other conditions mentioned occasionally included diet, family troubles, lifting heavy things, blood diseases (coagulation disorders), disturbed blood circulation in the legs, surgery, unhealthy lifestyle, sleep apnea syndrome, estrogens, caeliac disease.

After performing an ordered logistic regression analysis, we observed that higher age, having a diagnosis of cerebral haemorrhage (OR 0.318; 95% CI 0.129 - 0.783) or living alone (OR 0.490; 95% CI 0.241 - 0.997) were related to poorer knowledge of stroke/TIA risk factors. For patients who reported having a diagnosis of atrial fibrillation (OR 2.822; 95% CI 1.267 - 6.285) and who reported that they had a family history of cardiovascular disease (OR 1.871; 95% CI 1.040 - 3.367), we found higher levels of knowledge of stroke/TIA risk factors. The model was adjusted for age (Table 4).

**Table 4:** Model of factors influencing the knowledge about stroke/TIA risk factors (adjusted for age).

	Univariable	Multivariable		
Variable	OR	OR	95% CI	**p-value**^1)^
Age	0.971	-	-	-
Family history of cardiovascular disease	2.242	1.871	1.040 - 3.367	0.036*
Atrial fibrillation	2.478	2.822	1.267 - 6.285	0.011*
Diagnosis of cerebral haemorrhage	0.215	0.318	0.129 - 0.783	0.013*
Living alone	0.677	0.490	0.241 - 0.997	0.049*

The table shows a model including factors that significantly influenced the knowledge of stroke/TIA risk factors, adjusted for age. Knowledge of stroke/TIA risk factors is defined as the number of correctly identified factors in the questionnaire, categorized into 3 groups: good, moderate or poor knowledge. Odds ratio (OR) > 1 means that the factor is related to better knowledge, and OR < 1 to poorer knowledge. The univariable OR for the included variables are also given in the table.

1) Results from ordered logistic regression analysis; Statistical significance at level: *) p < 0.05; **) p < 0.01; ***) p < 0.001.

### Knowledge about treatment for reducing the risk of having a new stroke/TIA

When asked to list their drugs and to mark those that they thought were intended to prevent recurrent stroke/TIA events, 165 of the 182 patients (90.7%) reported taking drugs, 5 (2.7%) reported not taking any drugs, and 12 (6.6%) did not answer the question. Nearly half of the 165 patients who listed their drugs (86; 47.2%) did not mark any of them as preventive.

Anticoagulants and platelet aggregation inhibitors are important drugs for stroke/TIA prevention but only half of the patients who reported taking these drugs marked them as intended for prevention. About a third of patients who reported taking antihypertensive or antilipemic agents and very few of those taking hypoglycemic agents marked them as intended for prevention (Table 5).

**Table 5:** Patients' recognition of risk factor treatment.

Treatment	Number of patients who reported treatment n = 182 (%)	Number of those who marked the treatment as intended for stroke/TIA prevention n = first column (%)
Anticoagulants (warfarin)	32 (17.6)	18 (56.3)
Platelet aggregation inhibitors	114 (62.4)	55 (48.2)
Antihypertensive agents	120 (66.0)	43 (35.8)
Antilipemic agents	73 (40.1)	26 (35.6)
Hypoglycemic agents	21 (11.5)	3 (14.3)

Number and proportion of patients who reported taking drugs and listing them as intended for stroke/TIA prevention.

## Discussion

Our study showed that stroke/TIA patients' knowledge varied concerning risk factors for having a new stroke/TIA. Some diseases/conditions (hypertension and hyperlipidemia) and lifestyle related factors (smoking, absence of regular exercise, overweight) seemed to be quite well known stroke/TIA risk factors, both generally among all patients and among the patients who considered those risk factors as their own. Specific diseases such as carotid stenosis, atrial fibrillation and diabetes mellitus were identified as risk factors to a lesser extent. Patients who knew that they suffered from atrial fibrillation and carotid stenosis could, however, identify those conditions as stroke/TIA risk factors in more than 90% of cases. Kraywinkel et al also reported better knowledge of a specific risk factor among those affected by it [23].

Patients with atrial fibrillation seemed to have significantly better knowledge of stroke/TIA risk factors compared to the whole group of patients. This could be the result of more frequent contacts with medical care (because of regular monitoring of treatment with warfarin, for example), or a particular interest in health issues, since these patients had probably received more detailed information as the drugs they were taking have substantial risks of side effects.

Diabetes mellitus had the lowest identification rate. Furthermore, among patients who considered diabetes as a disease of their own, only 72.2% could identify diabetes as a stroke/TIA risk factor. Poor understanding of the fact that diabetes could be a stroke/TIA risk factor was observed previously by Kraywinkel et al in a study conducted among people without prior stroke/TIA events [23] and also in a study by Maasland et al [25]. In a study conducted in India, diabetes was among the best known stroke/TIA risk factors, at the same level as hypertension, smoking and excessive intake of alcohol, but the general level of knowledge was much lower than in our study [18]. In the prevention and education of the diabetic patients, most focus tend to be on cardiovascular complications and complications from the eyes and the feet, and cerebrovascular complications may receive less attention.

To our surprise, only 62% of the patients could identify the fact that a previous event of stroke/TIA was a factor that increased the risk of having a new stroke/TIA.

As in other studies [11], a number of patients suggested "stress" as a stroke/TIA risk factor. However, the relation between stress and stroke/TIA is still undetermined [26].

Patients in our study who had a registered diagnosis of cerebral haemorrhage had a lower level of knowledge about stroke/TIA risk factors, which could correspond to the generally worse clinical condition (including cognitive impairment) that follows a haemorrhagic stroke as compared to an ischaemic stroke [27]. Occasionally, a diagnosis of cerebral haemorrhage has also been used as an exclusion criteria when studying knowledge about risk factors [15].

In some studies, a number of sociodemographic factors have been found to influence patients' knowledge about stroke/TIA risk factors; for example, older age, male sex, and lower educational level, and this is in line with our results [28]. We found that being 75 years or older, or living alone, were factors that negatively influenced knowledge of stroke/TIA risk factors. Higher age was found to have a negative effect in some studies [11,14,15] but no effect in others [23]. Lower educational level was found to have a negative effect in a a study conducted in India [18], but not in some other studies [15,23].

The task of listing their drugs and marking those regarded as being prescribed to prevent new events of stroke/TIA seemed to be difficult, since less than half of the patients who listed their drugs marked any of them as intended to prevent stroke/TIA. Patients who are given anticoagulants or platelet aggregation inhibitors should be informed about the preventive effect of these drugs, and the fact that only 56.3% of those reporting the use of anticoagulants and 48.2% of those reporting the use of platelet aggregation inhibitors indicated that their purpose was preventive is worrying. The still lower proportions for patients using antihypertensive, antilipemic and hypoglycemic agents also indicate the importance of better patient education.

The fact that the patients who had attended the group meetings for stroke/TIA patients at GPHCC did not have better knowledge about risk factors than the rest of the group emphasizes the need for further studies regarding teaching strategies focusing on stroke/TIA patients.

There is a very strong tradition in Sweden and in the other Nordic countries to refer patients suspected of having suffered a stroke/TIA to hospital for Computed Tomography or Magnetic Resonance Imaging. National, regional and local guidelines stress the importance of referring all patients with recent (less than a week) symptoms compatible with stroke/TIA directly to hospital for further investigations and evaluation [29]. Recent Swedish studies show that only about five percent of first-ever stroke patients have not been in contact with hospital, and these comprise mainly patients living in nursing homes [30]. It is thus a strength of this study that we are likely to have included nearly all first-ever stroke/TIA patients. The patients we might have failed to include could be nursing home patients with concomitant severe diseases, or patients who never saw a doctor for their stroke/TIA symptoms.

One limitation of this study could be that we chose to design the questionnaire especially for the purpose of the study, as we could not find any questionnaires in the literature that were suitable. The questionnaire included a large number of close-ended questions, which made it rather long (eight pages). However, close-ended questions could result in a higher percentage of answers as compared to the open-ended questions used in several previous studies [23]. Although the response rate was rather good, the complexity and size of the questionnaire may have influenced it negatively. The fact that the risk factors were self reported makes misunderstandings possible. Another limitation is the small number of patients in some of the subgroups, for example patients with excessive alcohol consumption or carotid stenosis.

## Conclusions

Our study shows that knowledge about hypertension, hyperlipidemia and smoking as risk factors for new events of stroke/TIA was good, and that patients who suffered from atrial fibrillation or carotid stenosis seemed to be well informed about these conditions as risk factors. However, the level of knowledge was low regarding diabetes as a risk factor, and about the use of anticoagulants and platelet aggregation inhibitors for stroke/TIA prevention. Patients who had attended the group meetings did not have better knowledge than non-attendants. It thus seems necessary to develop individual teaching strategies for stroke/TIA patients, taking each patient's background into account. Special attention should be focused on diabetic patients to ensure that they understand that stroke/TIA can also be a consequence of diabetes.

## Competing interests

The authors declare that they have no competing interests.

## Authors' contributions

All authors have contributed to the conception and design of the study. AS carried out the data collection. All authors participated in the analysis and interpretation of the data and writing and approval of the final version of the manuscript.

## Pre-publication history

The pre-publication history for this paper can be accessed here:

http://www.biomedcentral.com/1471-2296/11/47/prepub

## References

[B1] Stegmayr B Asplund K Improved survival after stroke but unchanged risk of incidence Lakartidningen 2003 100 44 3492 3498 14651007

[B2] Johnson P Rosewell M James MA How good is the management of vascular risk after stroke, transient ischaemic attack or carotid endarterectomy? Cerebrovasc Dis 2007 23 2-3 156 161 10.1159/000097053 17124397

[B3] Clark TG Murphy MF Rothwell PM Long term risks of stroke, myocardial infarction, and vascular death in "low risk" patients with a non-recent transient ischaemic attack J Neurol Neurosurg Psychiatry 2003 74 5 577 580 10.1136/jnnp.74.5.577 12700296 PMC1738460

[B4] Sacco RL Wolf PA Kannel WB McNamara PM Survival and recurrence following stroke. The Framingham study Stroke 1982 13 3 290 295 7080120 10.1161/01.str.13.3.290

[B5] Glader EL Stegmayr B Norrving B Terent A Hulter-Asberg K Wester PO Asplund K Large variations in the use of oral anticoagulants in stroke patients with atrial fibrillation: a Swedish national perspective J Intern Med 2004 255 1 22 32 10.1046/j.0954-6820.2003.01253.x 14687235

[B6] Girot M Mackowiak-Cordoliani MA Deplanque D Henon H Lucas C Leys D Secondary prevention after ischemic stroke. Evolution over time in practice J Neurol 2005 252 1 14 20 10.1007/s00415-005-0591-8 15654550

[B7] Croquelois A Bogousslavsky J Risk awareness and knowledge of patients with stroke: results of a questionnaire survey 3 months after stroke J Neurol Neurosurg Psychiatry 2006 77 6 726 728 10.1136/jnnp.2005.078618 16549417 PMC2077461

[B8] O'Mahony PG Rodgers H Thomson RG Dobson R James OF Satisfaction with information and advice received by stroke patients Clin Rehabil 1997 11 1 68 72 10.1177/026921559701100110 9065362

[B9] Blades LL Oser CS Dietrich DW Okon NJ Rodriguez DV Burnett AM Russell JA Allen MJ Fogle CC Helgerson SD Rural community knowledge of stroke warning signs and risk factors Prev Chronic Dis 2005 2 2 A14 15888225 PMC1327708

[B10] Ferris A Robertson RM Fabunmi R Mosca L American Heart Association and American Stroke Association national survey of stroke risk awareness among women Circulation 2005 111 10 1321 1326 10.1161/01.CIR.0000157745.46344.A1 15769775

[B11] Kothari R Sauerbeck L Jauch E Broderick J Brott T Khoury J Liu T Patients' awareness of stroke signs, symptoms, and risk factors Stroke 1997 28 10 1871 1875 9341687 10.1161/01.str.28.10.1871

[B12] Pancioli AM Broderick J Kothari R Brott T Tuchfarber A Miller R Khoury J Jauch E Public perception of stroke warning signs and knowledge of potential risk factors Jama 1998 279 16 1288 1292 10.1001/jama.279.16.1288 9565010

[B13] Gupta A Thomas P General perception of stroke. Knowledge of stroke is lacking Bmj 2002 325 7360 392 10.1136/bmj.325.7360.392/a 12183323 PMC1123902

[B14] Samsa GP Cohen SJ Goldstein LB Bonito AJ Duncan PW Enarson C DeFriese GH Horner RD Matchar DB Knowledge of risk among patients at increased risk for stroke Stroke 1997 28 5 916 921 9158625 10.1161/01.str.28.5.916

[B15] Koenig KL Whyte EM Munin MC O'Donnell L Skidmore ER Penrod LE Lenze EJ Stroke-related knowledge and health behaviors among poststroke patients in inpatient rehabilitation Arch Phys Med Rehabil 2007 88 9 1214 1216 10.1016/j.apmr.2007.05.024 17826471

[B16] Clark MS Smith DS Knowledge of stroke in rehabilitation and community samples Disabil Rehabil 1998 20 3 90 96 10.3109/09638289809166061 9548020

[B17] Stein J Shafqat S Doherty D Frates EP Furie KL Patient knowledge and expectations for functional recovery after stroke Am J Phys Med Rehabil 2003 82 8 591 596 10.1097/00002060-200308000-00004 12872015

[B18] Das K Mondal GP Dutta AK Mukherjee B Mukherjee BB Awareness of warning symptoms and risk factors of stroke in the general population and in survivors stroke J Clin Neurosci 2007 14 1 12 16 10.1016/j.jocn.2005.12.049 17092722

[B19] Forster A Smith J Young J Knapp P House A Wright J Information provision for stroke patients and their caregivers Cochrane Database Syst Rev 2001 3 CD001919 11687003 10.1002/14651858.CD001919

[B20] Socialstyrelsen Klassifikation av sjukdomar och hälsoproblem 1997. Primärvård. Version KSH97-P 1996

[B21] Herrmann N Black SE Lawrence J Szekely C Szalai JP The Sunnybrook Stroke Study: a prospective study of depressive symptoms and functional outcome Stroke 1998 29 3 618 624 9506602 10.1161/01.str.29.3.618

[B22] Sug Yoon S Heller RF Levi C Wiggers J Fitzgerald PE Knowledge of stroke risk factors, warning symptoms, and treatment among an Australian urban population Stroke 2001 32 8 1926 1930 11486127 10.1161/01.str.32.8.1926

[B23] Kraywinkel K Heidrich J Heuschmann PU Wagner M Berger K Stroke risk perception among participants of a stroke awareness campaign BMC Public Health 2007 7 39 10.1186/1471-2458-7-39 17371603 PMC1838904

[B24] Hacke W Kaste M Bogousslavsky J Brainin M Chamorro A Lees K Leys D Kwiecinski H Toni P Langhorne P European Stroke Initiative Recommendations for Stroke Management-update 2003 Cerebrovasc Dis 2003 16 4 311 337 10.1159/000072554 14584488

[B25] Maasland L Koudstaal PJ Habbema JDF Dippel DWJ Knowledge and Understanding of Disease Process, Risk Factors and Treatment Modalities in Patients with a Recent TIA or Minor Ischemic Stroke Cerebrovasc Dis 2007 23 435 440 10.1159/000101468 17406114

[B26] Truelsen T Bonita R Advances in ischemic stroke epidemiology Adv Neurol 2003 92 1 12 12760161

[B27] Ferro JM Canhao P Peralta R Update on subarachnoid haemorrhage J Neurol 2008 255 4 465 479 10.1007/s00415-008-0606-3 18357424

[B28] Nicol MB Thrift AG Knowledge of risk factors and warning signs of stroke Vasc Health Risk Manag 2005 1 2 137 147 10.2147/vhrm.1.2.137.64085 17315400 PMC1993942

[B29] VISS, Neurology, Stroke Guidelines for Stockholm County Council (in Swedish) 2002 http://www.viss.nu

[B30] Appelros P Högerås N Térent A Case ascertainement in stroke studies: the risk of selection bias Acta Neurol Scand 2003 107 145 149 10.1034/j.1600-0404.2003.02120.x 12580866

